# Comparative outcomes of robotic single-port vNOTES versus robotic-Xi assisted single-incision transumbilical surgery for hysterectomy

**DOI:** 10.3389/fsurg.2026.1820180

**Published:** 2026-05-08

**Authors:** Qiannan Yang, David Pasternak, Chunhua Zhang, Xiaoming Guan

**Affiliations:** 1 Division of Minimally Invasive Gynecologic Surgery, Baylor College of Medicine, Houston, TX, United States; 2 Department of Obstetrics and Gynecology, The Second Affiliated Hospital of Nanjing Medical University, Nanjing, Jiangsu, China

**Keywords:** hysterectomy, robotic single port surgery, robotic-assisted surgery, single-incision transumbilical surgery (SITS), transvaginal natural orifice transluminal endoscopic surgery (vNOTES)

## Abstract

**Introduction:**

This study was to evaluate the safety and feasibility of robotic single-port transvaginal natural orifice transluminal endoscopic surgery (RSP-vNOTES) compared with robotic Xi-assisted single-incision transumbilical surgery (RXi-SITS) for hysterectomy.

**Method:**

A total of 405 patients was retrospectively analyzed, comprising 278 individuals who underwent RXi-SITS hysterectomy and 127 who underwent RSP-vNOTES hysterectomy.

**Results:**

A significantly higher prevalence of endometriosis-associated concomitant procedures, including ovarian cystectomy, lesion excision, adhesiolysis, appendectomy, bowel shaving, and bowel oversew, was observed among patients undergoing RSP-vNOTES (all *p* < 0.05). The RXi-SITS group saw uterosacral ligament suspension and ovarian vein ligation more commonly performed (both *p* < 0.05). A multivariable linear regression approach was employed to account for heterogeneity in procedural complexity. Following multivariable adjustment, estimated blood loss, hysterectomy time, and postoperative pain outcomes were comparable between the two surgical approaches. In contrast, multivariable linear regression accounting for concomitant procedures demonstrated that the RXi-SITS approach was independently associated with a significantly prolonged total operative time, with an adjusted mean increase of 21.8 min compared with RSP-vNOTES (B = 21.83; 95% CI: 6.05-37.61; *p* = 0.007). This is despite unadjusted analyses showing a longer median operative time in the RSP-vNOTES group (160 [IQR 140-194] vs. 151 [120–200] minutes; *p* = 0.02).

**Discussion:**

Compared with RXi-SITS hysterectomy, RSP-vNOTES hysterectomy demonstrated comparable surgical outcomes, supporting its safety and feasibility across a range of gynecologic procedures. Despite greater procedural complexity in the RSP-vNOTES cohort, the RXi-SITS approach was independently associated with a longer total operative time, suggesting a potential efficiency advantage of RSP-vNOTES in appropriately selected patients.

## 1 Introduction

Robotic surgical platforms were developed to mitigate the intrinsic technical limitations of conventional laparoscopy through the integration of tremor suppression, three-dimensional high-definition visualization, and wristed instrumentation (1). In recent years, robot-assisted surgical approaches have been increasingly integrated into clinical practice, offering enhanced dexterity and ergonomic advantages over traditional laparoscopy that may translate into improved surgical performance and outcomes (2, 3). By allowing true single-port instrumentation and facilitating natural orifice techniques, the da Vinci SP system represents an important evolution in minimally invasive robotic surgery. In the United States, the da Vinci SP platform has received regulatory authorization for clinical application in multiple specialties, including colorectal surgery, urology, thoracic surgery, and transoral otolaryngologic procedures (4–6). Regulatory approval for gynecologic applications has not yet been granted; nevertheless, the da Vinci SP robotic platform has been utilized for single-port gynecologic procedures in an investigation and research context.

Hysterectomy represents one of the most prevalent gynecologic surgeries performed in the United States, with approximately 500,000–600,000 procedures undertaken annually, ranking it second only to cesarean delivery among women's surgical interventions (7, 8). Within robotic surgical systems, hysterectomy may be performed using either single-port or multi-port da Vinci platforms, with access achieved through transabdominal or transvaginal approaches (9–12). Prior comparative investigation of robotic transvaginal natural orifice transluminal endoscopic surgery has demonstrated that both robotic multi-port vNOTES (RMP-vNOTES) and robotic single-port vNOTES (RSP-vNOTES) are safe and effective approaches for hysterectomy. RSP-vNOTES has been reported to offer logistical and ergonomic advantages while enabling the execution of technically complex procedures, including extensive endometriosis resection (13). Prior comparative studies indicate that robotic single-port hysterectomy is a feasible alternative to multi-port robotic hysterectomy (13). These studies showed that differences in operative time were influenced by surgeon experience and learning-curve effects, while perioperative morbidity was more strongly linked to case complexity and patient-related factors than to the specific robotic platform; notably, in high-experience settings, skills acquired with multi-port systems appear to transfer effectively to the da Vinci SP platform (14, 15). Currently, direct comparisons of surgical outcomes between RSP-vNOTES hysterectomy and robotic Xi-assisted single-incision transumbilical surgery (RXi-SITS) hysterectomy have not been reported. Accordingly, this study was designed to evaluate and compare perioperative outcomes of RSP-vNOTES and RXi-SITS hysterectomy performed for benign gynecologic indications.

## 2 Materials and methods

### 2.1 Study design

This study employed a retrospective cohort design, with surgical approach (RSP-vNOTES or RXi-SITS hysterectomy) defined as the primary exposure. Patients undergoing either surgical approach between January 2015 and November 2025 were identified through the primary surgeon's operative case log. All operations were completed by one specialized minimally invasive gynecologic surgeon practicing at two Baylor College of Medicine-affiliated institutions in Houston, Texas.

Eligible patients included women undergoing hysterectomy for benign gynecologic indications using either RSP-vNOTES or RXi-SITS. To ensure comparability between cohorts, patients requiring concurrent non-gynecologic procedures-such as segmental bowel resection, partial bladder resection for deep infiltrating endometriosis, or thoracic procedures for extra-pelvic disease-were excluded from both groups.

Baseline demographic variables included age, body mass index (BMI), race, prior vaginal delivery, history of cervical or abdominal surgery, and tobacco use. Perioperative outcomes included estimated blood loss, hysterectomy time (defined as the interval from colpotomy initiation to complete uterine detachment), total operative time (defined as the interval from skin incision or vaginal colpotomy to surgical closure), robotic docking time, and conversion rate. Postoperative outcomes included uterine specimen weight, same-day discharge rate, and patient-reported pain scores measured using a 0–10 visual analog scale (VAS) at postoperative weeks 1, 2, and 3. Standardized pain assessment was implemented in July 2018; therefore, earlier cases lacked uniform pain data and were excluded from pain analyses. Intraoperative complications (including bladder, bowel, and ureteral injuries) and postoperative complications within six weeks were recorded and classified according to the Clavien-Dindo grading system.

### 2.2 Operative technique

Selection of the hysterectomy approach was based on the primary surgeon's standard clinical practice during the study period. From January 2015 through June 2019, hysterectomies were performed exclusively using the RXi-SITS approach. Beginning in June 2019, RMP-vNOTES hysterectomy utilizing the da Vinci Xi platform was incorporated into clinical practice. Subsequently, from November 2023 through November 2025, RSP-vNOTES hysterectomy performed with the da Vinci SP platform became the predominant surgical approach.

#### 2.2.1 RXi-SITS hysterectomy

**Figure 1:**
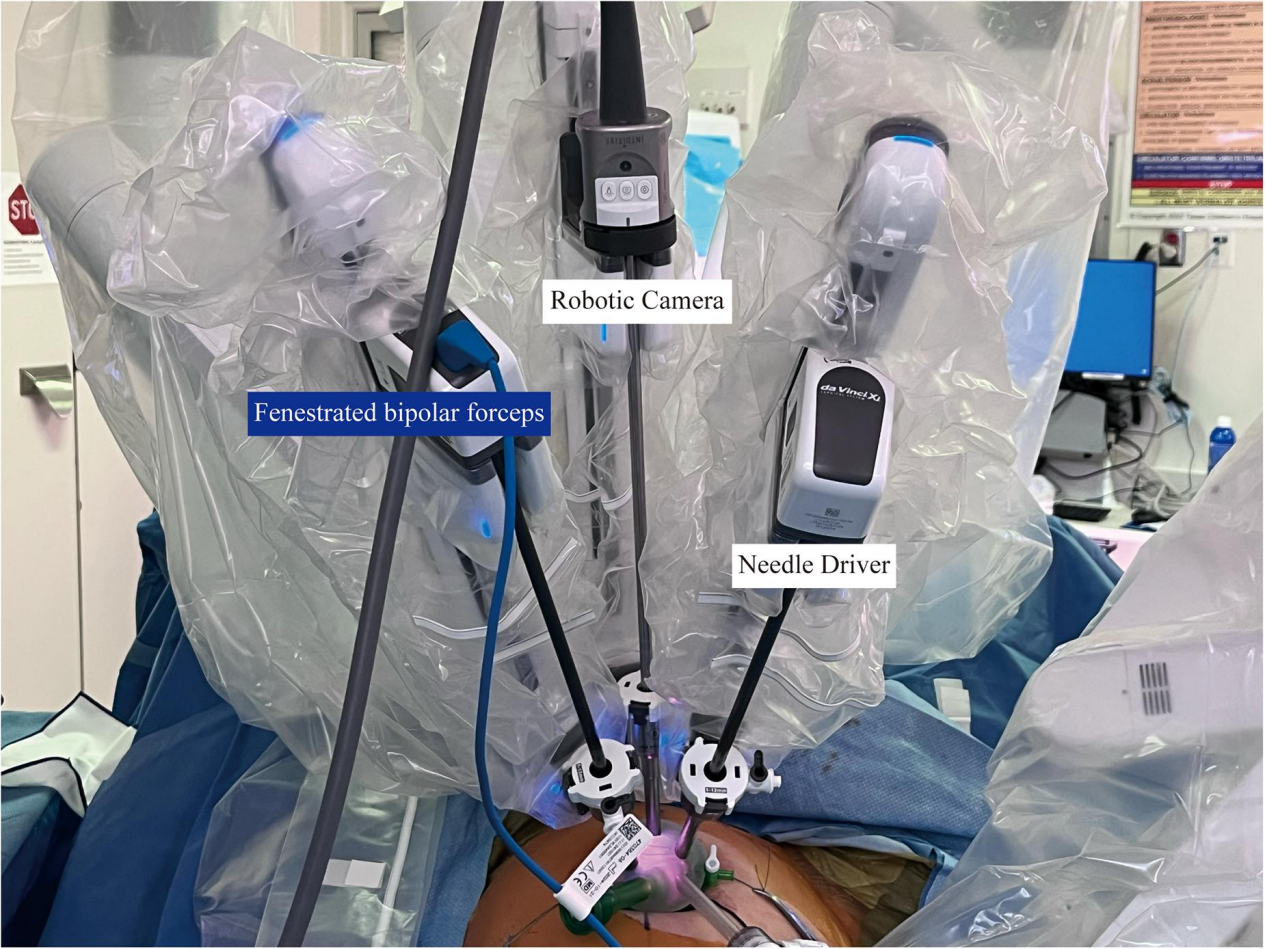
Da vinci Xi port placement using a five-lumen single-site access port for RXi-SITS.

Patients were placed in the dorsal lithotomy position. A RUMI II uterine manipulator with a Koh-Efficient colpotomy cup (Cooper Surgical, Trumbull, CT, USA) was utilized. A 25-mm transumbilical incision was created, followed by placement of an Alexis wound retractor and a five-lumen single-site access port. The port accommodated a AirSeal® insufflation channel, an endoscope, and two instruments channel. The da Vinci Xi system was docked (Figure 1), and hysterectomy was performed using standard robotic techniques. Concomitant procedures were performed as indicated. Vaginal cuff closure was completed robotically using a continuous barbed suture. The robotic system was then undocked, and the umbilical incision was closed in layers.

#### 2.2.2 RSP-vNOTES hysterectomy

**Figure 2:**
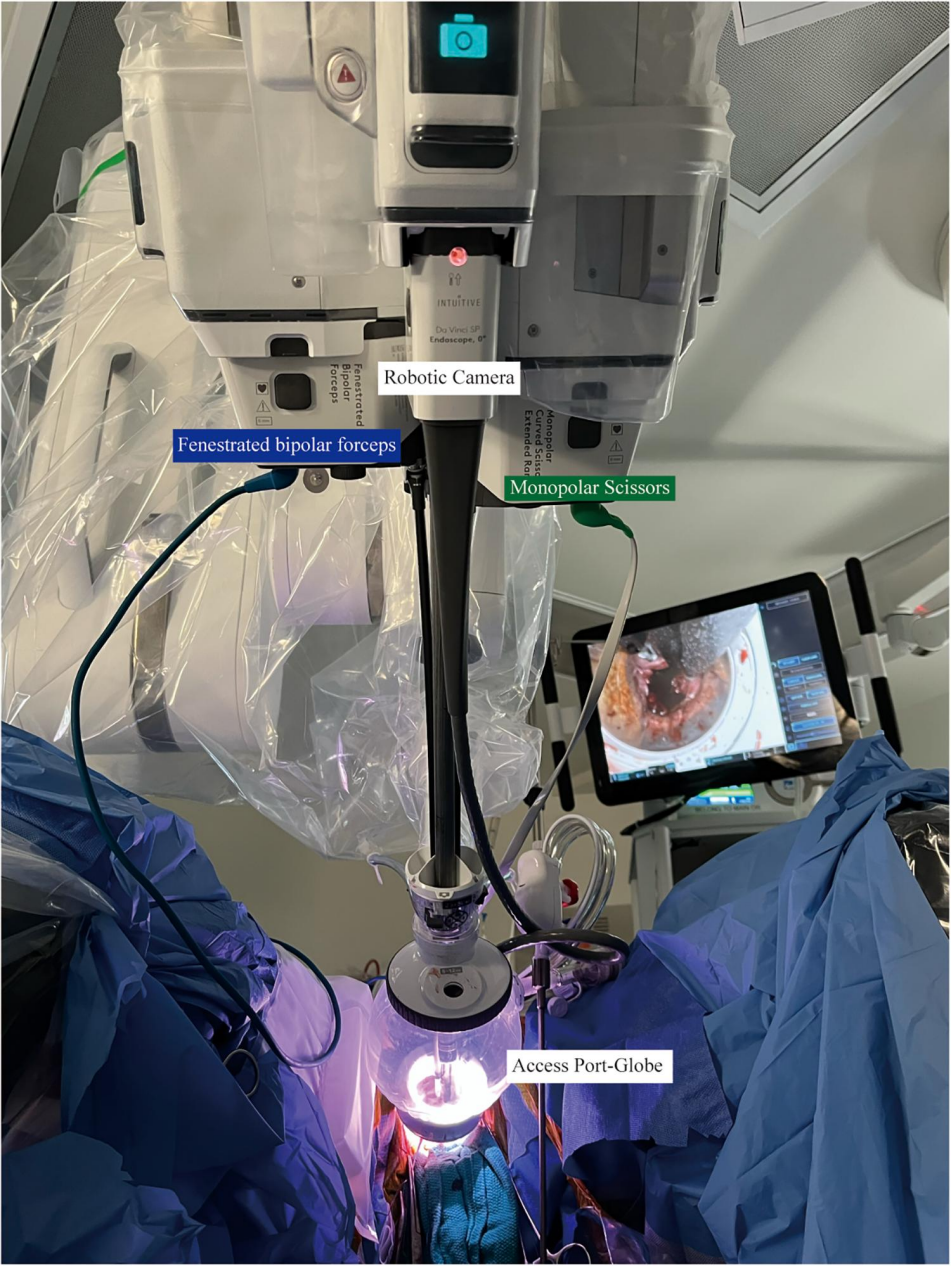
Da vinci SP port placement demonstrating docking of the robotic arm to the SP access platform (SP Globe) for RSP-vNOTES.

The surgical technique for RSP-vNOTES hysterectomy was performed using a standardized approach. Surgery began with customary vaginal hysterectomy maneuvers, incorporating bilateral uterine artery pedicle ligation and transection (16). Upon completion of the preliminary operative steps, the da Vinci SP access port kit was placed to facilitate robotic docking and affixed to the vaginal cuff utilizing the “4-P” anchoring method (Figure 2) (17). After placement of the access port, robotic docking was completed, and hysterectomy and all required concomitant procedures were subsequently carried out with robotic assistance. Upon completion of the operation, hemostasis was ensured, the robotic platform was disengaged, and manual closure of the vaginal cuff was performed.

### 2.3 Data analysis

All statistical analyses were carried out using SPSS software (version 25.0; IBM Corp., Chicago, IL, USA), and statistical significance was defined by a *p* value of less than 0.05. Distributional properties of continuous variables were evaluated using the Kolmogorov–Smirnov test. As the primary outcome measures demonstrated nonparametric distributions, continuous variables were summarized using medians and interquartile ranges (IQR), and intergroup comparisons were conducted with the Mann–Whitney U test. Categorical data were summarized as counts with corresponding proportions and analyzed using Pearson's chi-square or Fisher's exact tests, selected based on cell distribution. To account for differences in surgical complexity, multivariable linear regression models were constructed adjusting for clinically relevant covariates, including concomitant procedures (e.g., endometriosis excision, adhesiolysis, and ovarian cystectomy).

## 3 Results

During the study interval, 405 individuals fulfilled eligibility requirements and underwent hysterectomy via either the RXi-SITS or RSP-vNOTES technique. The RXi-SITS approach accounted for 278 cases performed between June 2015 and July 2025, whereas 127 cases were completed using the RSP-vNOTES method from November 2023 to November 2025.

Baseline patient demographics and clinical variables for the study cohorts are presented in Table 1. Comparative analysis revealed no significant between-group differences in age, BMI, race, tobacco exposure, prior vaginal delivery, previous abdominal or cervical procedures, or uterine specimen weight.

**Table 1:** Comparison of baseline patient profiles between study cohorts (*N* = 405).

Variables	RSP-vNOTES (*N* = 127)	RXi-SITS (*N* = 278)	*p* value
Age, years	39 [35–44]	41 [35–46]	0.30
Body mass index, kg/m^2^	27 [23–31]	27 [23–32]	0.70
Race and ethnicity			0.54
Caucasian	83 (65.4)	167 (60.1)	
African American	20 (15.7)	40 (14.4)	
Asian	11 (8.7)	32 (11.5)	
Hispanic	13 (10.2)	39 (14.0)	
Tobacco use	15 (11.8)	23 (8.3)	0.26
Vaginal delivery history	47 (37.0)	115 (41.4)	0.41
Abdominal surgery history	100 (78.7)	220 (79.1)	0.93
Cervix surgery history	13 (10.2)	36 (12.9)	0.44
Uterine weight, g	98 [65–153]	111 [75–180]	0.11

RSP-vNOTES, robotic single port vaginal natural orifice transluminal endoscopic surgery; RXi-SITS, robotic-Xi assisted single-incision transumbilical surgery.

Continuous measures are summarized using median [IQR], while categorical variables are reported as number (percentage). Group comparisons were conducted using nonparametric or categorical tests, including the Mann–Whitney U test and Fisher's exact test or Pearson's chi-square, as appropriate.

Table 2 illustrates additional procedures performed at the time of hysterectomy. Endometriosis-associated interventions were more prevalent in the RSP-vNOTES group, including ovarian cystectomy, excision of endometriosis, adhesiolysis, appendectomy, bowel shaving, and bowel oversewing (all *p* < 0.05). Conversely, high uterosacral ligament suspension (HUSS) and ovarian vein ligation were conducted more often in the RXi-SITS cohort (both *p* < 0.05).

**Table 2:** Comparison of concomitant surgical procedures between cohorts (*N* = 405).

Additional surgical procedures	RSP-vNOTES (*N* = 127)	RXi-SITS (*N* = 278)	*p* value
US or BS or USO or BSO	121 (95.3)	271 (97.5)	0.24
Ovarian Cystectomy	55 (43.3)	42 (15.1)	0.001
USLS	8 (6.3)	79 (28.4)	0.001
Endometriosis excision	113 (89.0)	139 (50.0)	0.001
Lysis of adhesions	96 (75.6)	113 (40.6)	0.001
Appendectomy	22 (17.3)	3 (1.1)	0.001
Bowel shaving	29 (22.8)	26 (9.4)	0.001
Bowel oversew	17 (13.4)	11 (4.0)	0.001
Ovarian vein ligation	2 (1.6)	17 (6.1)	0.045
Transobturator sling	2 (1.6)	8 (2.9)	0.73
Sacrocolpopexy	0	6 (2.2)	0.18

RSP-vNOTES, robotic single port vaginal natural orifice transluminal endoscopic surgery; RXi-SITS, robotic-Xi assisted single-incision transumbilical surgery.

Data are expressed as number (percentage), with comparisons performed using Fisher's exact test or Pearson's chi-square test, as indicated.

Table 3 summarizes operative and postoperative outcomes. Median total operative time differed between groups, with longer operative times observed in the RSP-vNOTES cohort compared with the RXi-SITS cohort (160 vs. 151 min; *p* = 0.02). In contrast, the RSP-vNOTES approach was associated with shorter robotic docking time (2 vs. 3 min, *p* = 0.04) and lower estimated blood loss (25 vs. 50 mL, *p* = 0.03); although statistically significant, these differences were considered clinically minor. Same-day discharge occurred more frequently following RSP-vNOTES (90.6% vs. 78.8%, *p* = 0.004). No statistically significant between-group differences were identified in hysterectomy time, conversion to another surgical approach, or postoperative pain scores.

**Table 3:** Comparison of surgical outcomes between study cohorts (*N* = 405).

Variables	RSP-vNOTES (*N* = 127)	RXi-SITS (*N* = 278)	*p* value
Total operative time, min	160 [140–194]	151 [120–200]	0.02
Hysterectomy time, min	38 [26–50]	35 [25–52]	0.37
Robot docking time, min	2 [2–3]	3 [2–4]	0.04
Estimated blood loss, mL	25 [25–50]	50 [25–94]	0.03
Same-day discharge	115 (90.6)	212 (76.3)	0.001
Conversion	0	3 (1.1)	0.56
Pain score (week 1)	6 [4–8]	7 [5–8]	0.87
Pain score (week 2)	5 [2–6]	5 [3–6]	0.42
Pain score (week 3)	3 [1–5]	3 [1–4]	0.20

RSP-vNOTES, robotic single port vaginal natural orifice transluminal endoscopic surgery; RXi-SITS, robotic-Xi assisted single-incision transumbilical surgery.

Continuous measures are summarized using median [IQR], while categorical variables are reported as number (percentage). Group comparisons were conducted using nonparametric or categorical tests, including the Mann–Whitney U test and Fisher’s exact test or Pearson's chi-square, as appropriate.

In the multivariable linear regression model controlling for concomitant procedures, patients in the RXi-SITS group (group 1) had a significantly longer total operative time compared with the RSP-vNOTES group (group 0) (B = 21.83 min; 95% CI: 6.05–37.61; *p* = 0.007), indicating an average increase of approximately 22 min associated with the RXi-SITS approach. Hysterectomy time, intraoperative blood loss, and postoperative pain scores were comparable between the two cohorts (Table 4).

**Table 4:** Results of multivariable linear regression analysis.

Outcome	*Β* Coefficient	*p* value	95% CI for B	
			Lower bound	Upper bound
Total operative time, min	21.833	0.007	6.053	37.612
Hysterectomy time, min	−0.652	0.825	−6.444	5.140
Estimated blood loss, mL	20.887	0.073	−1.989	43.762
Pain score (week 1)	−0.091	0.785	−0.75	0.568
Pain score (week 2)	−0.469	0.207	−1.201	0.262
Pain score (week 3)	−0.541	0.113	−1.213	0.130

Adjustments were made for additional procedures (ovarian cystectomy, endometriosis excision, uterosacral ligament suspension, lysis of adhesions, bowel shaving, appendectomy, bowel oversew, ovarian vein ligation).

Three intraoperative conversions were recorded in the RXi-SITS group. One case required conversion to robot-assisted multi-port surgery due to bilateral distal ureteral obstruction, necessitating bilateral extravesical ureteroneocystotomy with right psoas hitch and placement of bilateral 6F × 26 cm ureteral stents by urology. The remaining two conversions were due to full-thickness rectal injuries sustained during adhesiolysis, both of which required open primary repair by general surgery. No conversions were observed in the RSP-vNOTES group.

Perioperative complications are summarized in Table 5. Overall complication rates were comparable between the two groups (15.0% in RSP-vNOTES vs. 15.5% in RXi-SITS, *p* = 0.90). Intraoperative events were uncommon in both cohorts, occurring in 1 patient (0.8%) in the RSP-vNOTES group and 8 patients (2.9%) in the RXi-SITS group (*p* = 0.28). Clavien-Dindo grade I-II complications were recorded in 11 patients (8.7%) following RSP-vNOTES and 30 patients (10.8%) following RXi-SITS (*p* = 0.51), with urinary tract infection being the most frequent in both groups. CD grade III events requiring reoperation occurred in 7 patients (5.5%) in the RSP-vNOTES cohort and 5 patients (1.8%) in the RXi-SITS cohort (*p* = 0.06). There were no observed grade IV or V complications in either study group.

**Table 5:** Perioperative complications by study cohort (*N* = 405).

Complications	RSP-vNOTES (*N* = 127)	RXi-SITS (*N* = 278)	*p* value
Intraoperative complications	1 (0.8)	8 (2.9)	0.28
Bladder injury	1 (0.8)	2 (0.7)	
Ureter injury	0	2 (0.7)	
Bowel injury	0	4 (1.4)	
CD grade I-II	11 (8.7)	30 (10.8)	0.51
Urinary tract infection	8 (6.3)	17 (6.1)	
Superficial surgical site infection	2 (1.6)	8 (2.9)	
Deep surgical site infection	1 (0.8)	1 (0.4)	
Venous thromboembolism	0	4 (1.4)	
CD grade III (Reoperation)	7 (5.5)	5 (1.8)	0.06
Vaginal cuff reoperation	3 (2.4)	1 (0.4)	
Pelvic abscess/fluid drainage	2 (1.6)	3 (1.1)	
Diagnostic laparoscopy	2 (1.6)	1 (0.4)	
Total complications	19 (15.0)	43 (15.5)	0.90

RSP-vNOTES, robotic single port vaginal natural orifice transluminal endoscopic surgery; RXi-SITS, robotic-Xi assisted single-incision transumbilical surgery.

Data are expressed as number (percentage), with comparisons performed using Fisher’s exact test or Pearson’s chi-square test, as indicated.

## 4 Discussion

The surgical management of benign gynecologic conditions has evolved toward the routine use of minimally invasive hysterectomy approaches (18). As both surgeons and patients increasingly prioritize cosmetic outcomes, there has been a progressive shift from multi-port to single-port approaches. This includes the adaptation of single-port techniques using robotic multi-port platforms-the da Vinci Xi system (19). With advances in surgical technology, the da Vinci SP system has been developed as a platform specifically designed for single-port procedures. The system employs a single robotic arm introduced through a solitary incision, incorporating an articulating three-dimensional camera and three fully wristed, multi-articulated instruments. This configuration provides dual internal articulation points, facilitating enhanced triangulation and improved access to the operative field (20). In this comparative analysis, RSP-vNOTES hysterectomy demonstrated perioperative outcomes comparable to RXi-SITS hysterectomy, despite a higher frequency of complex concomitant procedures in the RSP-vNOTES cohort. After adjustment for procedural complexity, the RXi-SITS approach was independently linked to increased total operative time, while blood loss, hysterectomy time, and postoperative pain were similar between groups. These results are consistent with prior reports demonstrating the safety and effectiveness of robotic vNOTES approaches for hysterectomy. While earlier studies have compared RSP-vNOTES with RXi-vNOTES or traditional laparoscopic-vNOTES, direct comparisons with RXi-SITS hysterectomy have been lacking (13, 21). Our study addresses this gap and provides comparative data across two commonly utilized robotic platforms.

Although unadjusted analyses suggested longer operative time in the RSP-vNOTES cohort, this association was attenuated and reversed after adjustment for concomitant procedures, highlighting the influence of case complexity. The RSP-vNOTES group included a higher proportion of additional procedures, and in the multivariable model, bowel procedures (bowel oversewing and shaving), adhesiolysis, and ovarian cystectomy were significantly associated with increased operative duration. These complexity-related factors likely contributed to the longer operative times observed in the unadjusted analysis. After accounting for these variables, the independent effect of surgical approach became more apparent, with longer adjusted operative times observed in the RXi-SITS group. This finding underscores the importance of accounting for case mix in observational studies, as unadjusted comparisons may be confounded by differences in surgical complexity. Collectively, these results suggest that operative time is driven not only by surgical approach but also by the underlying procedural burden. The longer adjusted operative time associated with RXi-SITS may reflect intrinsic differences in surgical workflow, including the transumbilical access route, port configuration, instrument exchange, and docking processes. In contrast, the transvaginal access utilized in RSP-vNOTES avoids the need for abdominal incision closure, which may further contribute to procedural efficiency. These findings are consistent with prior studies demonstrating comparable operative times between RSP-vNOTES and other minimally invasive approaches, as well as reports of longer operative duration in transumbilical single-site robotic surgery (13, 22, 23).

The higher frequency of endometriosis-related interventions observed in the RSP-vNOTES cohort highlights the feasibility of this approach for managing complex pelvic pathology. However, the lower proportion of such procedures in the RXi-SITS group should not be interpreted as a limitation of the RXi-SITS technique. Indeed, RXi-SITS is well suited for managing advanced disease requiring extensive upper abdominal or extra-pelvic interventions, such as bowel resection for deep infiltrating endometriosis or diaphragmatic endometriosis excision, procedures that were not included in the present analysis given their relative rarity and limited feasibility via a transvaginal approach (24, 25). The observed differences in concomitant procedures between groups-including a higher frequency of endometriosis-related interventions in the RSP-vNOTES cohort and increased rates of uterosacral ligament suspension and ovarian vein ligation in the RXi-SITS cohort may reflect temporal shifts in the primary surgeon's clinical practice. Specifically, RXi-SITS hysterectomy was adopted earlier in the surgeon's practice (June 2015), during a period when surgical management was more commonly focused on pelvic organ prolapse and uterine fibroids. In contrast, the introduction of RSP-vNOTES occurred later (November 2023), coinciding with an increased emphasis on the surgical treatment of endometriosis, which likely contributed to the higher prevalence of endometriosis-related procedures in that group.

In the present study, the rate of complications was comparable between the two groups. All conversions occurred in the RXi-SITS group (3/278 = 1.1%) and were attributable to intraoperative complications or anatomic challenges necessitating necessitating open repair in 2 cases and conversion to multi-port in one case, whereas no conversions were observed in the RSP-vNOTES cohort. Although the overall conversion rate was low, this finding supports the technical feasibility of RSP-vNOTES in appropriately selected patients. These results are consistent with prior literature demonstrating low conversion rates in robot-assisted hysterectomy, including a large retrospective series reporting no conversions to laparotomy and only rare conversions (3/724 = 0.4%) to conventional laparoscopy, even in cases of increased surgical complexity (26). Similarly, a prior study comparing traditional laparoscopic and single-port robotic vNOTES hysterectomy reported no conversions in either group, further supporting the procedural feasibility of both approaches (27).

This study is subject to inherent limitations of its retrospective design, including potential selection bias and methodological constraints in data collection and analysis, as the choice of surgical approach was based on evolving clinical practice rather than predefined or randomized criteria; however, the comparable baseline characteristics between groups suggest a reasonable degree of clinical comparability. In addition, detailed postoperative pain data were unavailable for a subset of patients, as standardized pain questionnaires were introduced into clinical practice in July 2018, resulting in the exclusion of earlier RXi-SITS cases from pain score analyses. Furthermore, as the two cohorts were performed in different time periods, temporal bias related to evolving surgical expertise, perioperative care pathways, and institutional practices may be present. Although surgical experience generally improves over time, the adoption of a new robotic platform introduces a procedure-specific learning curve. Prior studies suggest that proficiency in RSP-vNOTES may require approximately 30 cases; therefore, early cases in this cohort may reflect platform-related learning rather than purely improved surgical efficiency, potentially offsetting the expected gains associated with temporal progression (28). All procedures were performed by a single highly experienced surgeon at a high-volume center, which reduces inter-operator variability and allows outcomes to be more directly attributed to the surgical platform; however, this may limit the generalizability of the findings to settings with varying levels of surgical expertise. Finally, although the relatively large cohort size and adjustment for concomitant procedures strengthen the overall validity of the findings, residual confounding related to underlying disease severity and surgical complexity, particularly in endometriosis cases, cannot be entirely excluded. Future prospective and randomized studies are warranted to validate these findings.

## 5 Conclusion

This study provides comparative evidence supporting the feasibility and safety of RSP-vNOTES hysterectomy relative to RXi-SITS across a range of gynecologic procedures. From a clinical perspective, these findings suggest that RSP-vNOTES may represent a viable alternative to RXi-SITS hysterectomy, even in patients requiring complex adjunctive procedures. Future investigations employing prospective designs and multicenter collaboration are needed to refine patient selection and evaluate long-term outcomes.

## Data Availability

The original contributions presented in the study are included in the article/Supplementary Material, further inquiries can be directed to the corresponding authors.
